# Targeting Future Pandemics, a Case for *De Novo* Purine Synthesis and Basic Research

**DOI:** 10.3389/fimmu.2021.694300

**Published:** 2021-06-11

**Authors:** Randall C. Mazzarino

**Affiliations:** Schepens Eye Research Institute of Mass Eye and Ear Infirmary and the Department of Ophthalmology, Harvard Medical School, Boston, MA, United States

**Keywords:** nucleotide synthesis, viral response, therapeutic development, immunology & infectious diseases, metabolism

## Abstract

We are currently experiencing a deadly novel viral pandemic with no efficacious, readily available anti-viral therapies to SARS-CoV-2. Viruses will hijack host cellular machinery, including metabolic processes. Here, I provide theory and evidence for targeting the host *de novo* purine synthetic pathway for broad spectrum anti-viral drug development as well as the pursuit of basic science to mitigate the risks of future novel viral outbreaks.

## Perspective

The SARS-CoV-2 pandemic is wreaking havoc across the world. Globally, case management has been mixed. Several countries are essentially beating the pandemic with ever decreasing case counts, while other countries are unable to contain the outbreak. Recent vaccine roll out has shown positive results in decreasing case counts. The threat of subsequent waves of infection is still a lurking fear for countries, indeed the World Health Organization has recently classified the India SARS-CoV-2 variant as a global health risk. Our preparedness for a novel viral pandemic was minimal, therapeutically speaking. While a vaccine is arguably the best course of action, this is not feasible during a novel pandemic as vaccines are virus strain specific and a novel viral outbreak represents an immediate need. The current SARS-CoV-2 pandemic has demonstrated that mRNA and adenovirus vector vaccines required approximately one year to develop, test, and introduce to the general population in response to a novel viral threat; hardly a time scale that represents an immediate response to a crisis. The mRNA vaccines have shown greater than 90% efficacy (1), which further cements that a vaccine is our greatest tool to stop the spread of a lethal virus. However at time of writing, there were a reported 160 million cases and 3.32 million deaths globally with approximately 32.8 million cases and 582,867 deaths within the United States, with peak single day death tolls between 3,000 and 4,000 (2). Novel virus outbreaks should not be considered a rare occurrence as recent history shows the ever looming threat; SARS-CoV, MERS-CoV, Zika, H7N9 (avian influenza), H1N1 (swine influenza), and others (3). Regarding coronaviruses, no antiviral drugs have been developed against the SARS-CoV, SARS-CoV-2, or MERS-CoV strains (4). The nucleoside analog RNA-dependent RNA Polymerase inhibitor Remdesivir was the only readily available drug with strong potential for treatment of SARS-CoV-2, with early evidence suggesting questionable success (5) however recently it has shown a viable treatment (6). Viruses are subject to evolutionary pressures and alterations in their genomic sequences causes variants to emerge; the developed vaccines efficacy to these new variants are currently in question, raising the possibilities of slightly reduced or unknown effectiveness (7) or the need for a multi-variant booster shot. It is therefore critical to develop and maintain a set of antiviral drugs so that efficient treatment may be implemented to rapidly address infections and consequent morbidity and mortality while a vaccine is being developed to a novel viral pathogen.

Antiviral development may be seen as targeting pathogens or the host; both have their benefits and detriments. Pathogen targeting therapeutics may inadvertently add pressure to select resistant mutant variants but also boasts greater effectiveness for the specific virus, while host targeting therapeutics do not add a mutation selecting pressure however will likely have a higher chance of patient side effects, including in those who are already immunocompromised. Broad spectrum antivirals should be seen as a viable option to blunt the initial consequences in a novel pandemic situation during which time pathogen specific therapeutics can be scrutinized and subsequently added as treatment options. Here I present rationale for targeting host cell metabolism, specifically the *de novo* purine synthesis enzyme ATIC, as a basis for broad spectrum antivirals as standalone or component in combination therapy, using the current SARS-CoV-2 pandemic as a viewing lens. I also draw on the successes of basic, blue sky research in finding host biological processes to be examined for novel areas of future therapeutic development.

The preponderance of evidence strongly suggests that morbidity and mortality from SARS-CoV-2 is mainly due to the inflammatory response or cytokine storm in response to infection (8–10). Positive outcomes regarding SARS-CoV-2 seem to be linked to a substantial CD4+ and CD8+ T cell response (11–14) however T cell exhaustion markers are noted in SARS-CoV-2 recovered patients (15). Recently, *in silico* modeling draws attention the Bradykinin response as a plausible explanation for SARS-CoV-2 morbidity and mortality as negative outcomes are often associated robust abnormal lung, cardiac, and vasculature responses (16). It should be noted, heightened immune and inflammatory responses to viral infections in general are common (17, 18) and amelioration of this response should be considered in development of broad-spectrum treatments.

**Figure 1:**
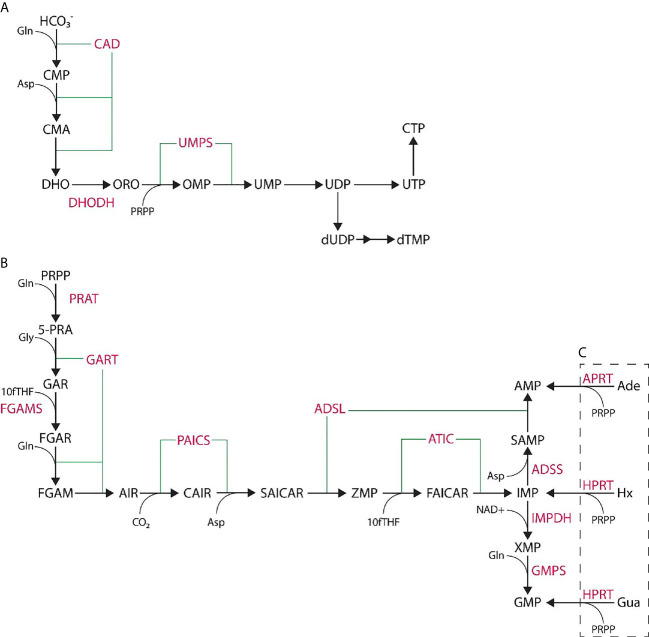
*De novo* nucleotide synthesis pathways. **(A)** *De novo* pyrimidine synthesis pathway produces UMP *via* six steps catalyzed by three enzymes and is further processed to UTP, CTP, or dTMP. **(B)** *De novo* purine synthesis pathway produces IMP *via* ten steps catalyzed by six enzymes. IMP is further processed to AMP or GMP through two more steps. **(C)** Purine salvage synthesis utilizes adenine (Ade), hypoxanthine (Hx), or guanine (Gua) to generate phosphorylated nucleotides in a single step. Enzymes noted in pink while small molecules are in black.

Purines and pyrimidines are essential small molecules that are the bases of nucleotides and are synthesized through either *de novo* or salvage pathways using small molecule precursors and cofactors (**Figure 1**). Salvage nucleotide synthesis utilizes a single reaction from component small molecules, such as hypoxanthine, adenine, or guanine for purine synthesis, or uracil, cytidine, or thymidine for pyrimidine synthesis which are typically derived from nucleotide catabolic products (e.g., nucleic acid turnover). *De novo* nucleotide synthesis utilizes more basic precursors: phosphoribosyl pyrophosphate (PRPP) for purine synthesis, and bicarbonate for pyrimidine synthesis. *De novo* purine synthesis (DNPS) requires ten sequential reactions catalyzed by six enzymes to form IMP, while *de novo* pyrimidine synthesis utilizes six sequential reactions catalyzed by three enzymes to form UMP (**Figure 1**). Salvage synthesis is energetically efficient and typically utilized as a maintenance system for existing nucleotide pools and energy whereas *de novo* synthesis upregulated at the G1/S phase interface to generate nucleotide pools necessary for genome replication (19–21). Purines perform multiple cellular functions such as energy currency, genomic information in RNA and DNA, signaling molecules, and cofactors.

Nucleotide pools represent an area of interest in viral therapeutic development, as the viral genome must be replicated to produce new viral particles from the infected host cell. Regarding the current pandemic, the SARS-CoV-2 genome is approximately 30 kilobase pairs of sense RNA, containing approximately 14 open reading frames. Host machinery translates viral mRNA to produce viral proteins which in turn creates organelles, transcribes viral RNA, and replicates its viral genome which are then packaged into viral progeny (22, 23). The proteins encoded by the viral genome promote its survival and virulence, and carry out its viral replication (23). This requirement for purine and pyrimidine nucleotides may be met *via* upregulation of *de novo* pathways through viral accessory protein interactions with host cell machinery (24, 25) making these pathways an ideal target; indeed a small number of antiviral drugs target nucleotide synthesis (26). Most all of the current therapeutic and research interest in *de novo* nucleotide synthesis is focused on pyrimidines. Most drugs targeting pyrimidine synthesis inhibit DHODH, which catalyzes the conversion of dihydroorotate to orotate. The cytidine analog Gemcitiabine inhibits pyrimidine synthesis (26) and has exhibited anti-inflammatory properties in a murine model (27). Indeed, pyrimidine synthesis inhibitors have been largely explored and have shown efficacy as broad-spectrum host targeting antiviral therapeutics as standalone (28, 29) and in cocktail with other antivirals (30). Drugs targeting purine synthesis largely inhibit the enzyme IMPDH (31), responsible for a step in the conversion of IMP to GMP, which is considered outside of *de novo* synthesis. Nucleotide targeting drugs have shown potency in treatment of both DNA and RNA viral infections (25). Indeed, targeting purine synthesis seems an ideal avenue as IMPDH inhibitors allow elevated proliferation levels of memory CD8+ T cells and show increased cytolytic activity over non-specific nucleotide or pyrimidine synthesis inhibitors (32).

Recent evidence suggests a major role for nucleotide products in the SARS-CoV-2 outbreak outside of nucleic acid synthesis. High dose methotrexate is hypothesized to be advantageous in treatment of severe SARS-CoV-2 cases (33, 34). Methotrexate is an inexpensive, widely available drug with potent anti-inflammatory properties acting as a folate synthesis and nucleotide (both purine and pyrimidine) synthesis inhibitor promoting intracellular accumulation of the *de novo* purine synthesis intermediate ZMP and increasing local concentrations of adenosine (35). The precise mechanism of action is unknown. In addition to inhibition of folate and nucleotide synthesis as well as decreasing inflammatory responses, it produces patient side effects, which suggests that it affects other biological processes (36). Methotrexate has, however, been found to hinder the cytolytic capabilities and clonal expansion of CD8+ memory T cells (32). Methotrexate may also compound complications with immunosuppressed individuals (e.g., cancer patients). Taken together, methotrexate may be an acceptable viral treatment for the time being (33) but it ideally should be replaced with newer therapeutics in the future. Antifolate derivatives (e.g., Lometrexol) are known to favorably target the trifunctional DNPS enzyme GART (37, 38), however exhibit potent cytotoxic properties (38). Due to this cytotoxic property of GART targeting antifolate drugs, these are not a likely viable option to further explore for viral response targeting.

ZMP is the first substrate for the bifunctional enzyme ATIC and is an AMP mimetic (39). ZMP can activate AMP-activated protein kinase (AMPK), a master regulator of cellular metabolism that has anti-inflammatory properties (40–42); indeed, a recent murine study showed that ZMP based AMPK activation inhibits and reverses the nuclear translocation of NFκB and reduces production of TNFα and IL1β (43). AMPK activates upon an increase in the AMP : ATP ratio and will inhibit ATP catabolic processes while upregulating ATP anabolic processes. DNPS is an energy intensive process, requiring five ATP molecules per one IMP synthesized. AMPK likely inhibits the *de novo* pathway and activates salvage pathways, energy production pathways such as glycolysis and fatty acid oxidation, as well as nucleotide kinase activities. One approach to inhibit DNPS and activate AMPK would be to target the ATIC enzyme *via* drug intervention. ATIC is only active as a homodimer; the small molecule Compound14 disrupts ATIC dimerization, halting *de novo* purine synthesis and promoting ZMP accumulation (44).

**Figure 2:**
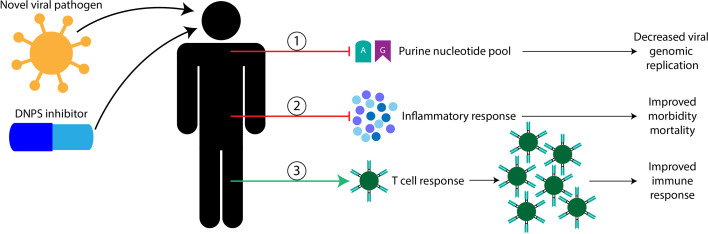
Proposed benefits of targeting host DNPS metabolism for novel viral pathogen infection. Here, I propose that host DNPS targeting therapeutics are likely to have three mechanisms of benefit, in no particular order. 1, DNPS inhibitors are likely to reduce purine nucleotide pools used to replicate the viral genome resulting in less viral proliferation. 2, DNPS inhibitors are likely to temper inflammatory responses reducing morbidity and mortality in severely affected patients. 3, DNPS inhibitors are likely permissive to T cell recognition and clonal expansion allowing natural host clearance of the viral infection.

My blue sky research was focused on characterizing the transcriptomes of three DNPS knock-out mutants [crGART, crADSL, and crATIC] (45). This research on dysregulation of DNPS revealed processes that are relevant to or directly involved in viral responses; given the importance of the nucleotide pool in viral propagation as outlined above this is perhaps unsurprising. Indeed, common themes include regulation of cell cycle, bradykinin systems (fluid shear stress, smooth muscle development and contraction, and cardiac terms) cytokine cascade terms (TNFα and various interleukins such as IL-1β, and others), interferon activity, inflammatory response, fatty acid processing (such as through arachidonic acid), as well as viral response terms such as, antiviral mechanism by IFN-stimulated genes and ISG15, OAS antiviral response, mast cell mediated immunity, T cell activation and differentiation, and CD4 and alpha-beta T cell activation (46–48). Targeting DNPS is thus likely to have multiple benefits (**Figure 2**): 1- reducing the production of nucleotides necessary for viral genome replication, 2- modulating the inflammatory response 3- allowing T cell proliferation and cytolytic function for natural host clearance of the viral infection. Taken together, DNPS may represent an ideal target for the development of new host targeted broad-spectrum antiviral drugs.

The basic research projects mentioned here were designed to identify cellular and organismal processes influenced by DNPS deficiency and pathway intermediate accumulation. Our research found changes relevant to many processes such as TGFβ signaling, neurodevelopment, inflammation, placental development, cell cycle regulation, in addition to processes involved in viral responses. Basic research has the benefit of identification of processes and applications previously unthought of or overlooked to areas of clinical relevance. This is seen with our identification of potential importance of DNPS to viral response, which were unexpected. Basic research therefore is necessary and should be actively pursued to identify areas that may prove fruitful in clinical applications.

## Data Availability Statement

The original contributions presented in the study are included in the article/supplementary material. Further inquiries can be directed to the corresponding author.

## Author Contributions

This perspective was developed, researched, and written by RM. All authors contributed to the article and approved the submitted version.

## Conflict of Interest

The author declares that the research was conducted in the absence of any commercial or financial relationships that could be construed as a potential conflict of interest.

## References

[1] Thomas K. New Pfizer Results: Coronavirus Vaccine Is Safe and 95% Effective. New York City, NY: New York Times (2020). Available at: https://www.nytimes.com/2020/11/18/health/pfizer-covid-vaccine.html. (Accessed November 18, 2020).

[2] Dong E Du H Gardner L. An Interactive Web-Based Dashboard to Track COVID-19 in Real Time. Lancet Inf Dis (2020) 20(5):533–4. 10.1016/S1473-3099(20)30120-1 PMC7159018 32087114

[3] World Health Organization. Disease Outbreaks by Year. Available at: https://www.who.int/csr/don/archive/year/en/.

[4] Gordon DE Jang GM Bouhaddou M Xu J Obernier K White KM. A SARS-CoV-2 Protein Interaction Map Reveals Targets for Drug Repurposing. Nature (2020) 583(7816):459–68. 10.1038/s41586-020-2286-9 PMC7431030 32353859

[5] Wang Y Zhang D Du G Du R Zhao J Jin Y. Remdesivir in Adults With Severe COVID-19: A Randomised, Double-Blind, Placebo-Controlled, Multicentre Trial. Lancet (2020) 395(10236):1569–78. 10.1016/S0140-6736(20)31022-9 PMC7190303 32423584

[6] Beigel JH Tomashek KM Dodd LE Mehta AK Zingman BS Kalil AC. Remdesivir for the Treatment of Covid-19 — Final Report. N Engl J Med (2020) 383(19):1813–26. 10.1056/NEJMc2022236 PMC7262788 32445440

[7] Callaway E Ledford H. How to Redesign COVID Vaccines So They Protect Against Variants. Nature (2021) 590(7844):15–6. 10.1038/d41586-021-00241-6 33514888

[8] Bradley BT Maioli H Johnston R Chaudhry I Fink SL Xu H. Histopathology and Ultrastructural Findings of Fatal COVID-19 Infections in Washington State: A Case Series. Lancet (2020) 396(10247):320–32. 10.1101/2020.04.17.20058545 PMC7365650 32682491

[9] Cao X. Covid-19: Immunopathology and its Implications for Therapy. Nat Rev Immunol (2020) 20(5):269–70. 10.1038/s41577-020-0308-3 PMC7143200 32273594

[10] Klok FA Kruip MJHA van der Meer NJM Arbous MS Gommers D Kant KM. Confirmation of the High Cumulative Incidence of Thrombotic Complications in Critically Ill ICU Patients With COVID-19: An Updated Analysis. Thromb Res (2020) 191:148–50. 10.1016/j.thromres.2020.04.041 PMC7192101 32381264

[11] Grifoni A Weiskopf D Ramirez SI Mateus J Dan JM Moderbacher CR. Targets of T Cell Responses to SARS-CoV-2 Coronavirus in Humans With COVID-19 Disease and Unexposed Individuals. Cell (2020) 181(7):1489–501.e15. 10.1016/j.cell.2020.05.015 32473127 PMC7237901

[12] Li J Wang J Kang AS Sacitharan PK. Mapping the T Cell Response to COVID-19. Signal Transduct Target Ther (2020) 5(1):112. 10.1038/s41392-020-00228-1 32616709 PMC7330543

[13] Qin C Zhou L Hu Z Zhang S Yang S Tao Y. Dysregulation of Immune Response in Patients With Coronavirus 2019 (COVID-19) in Wuhan, China. Clin Infect Dis (2020) 71(15):762–8. 10.1093/cid/ciaa248 PMC7108125 32161940

[14] Zhang H Wu T. Cd4+T, CD8+T Counts and Severe COVID-19: A Meta-Analysis. J Infect (2020) 81(3):e82–4. 10.1016/j.jinf.2020.06.036 PMC7305716 32569604

[15] Diao B Wang C Tan Y Chen X Liu Y Ning L. Reduction and Functional Exhaustion of T Cells in Patients With Coronavirus Disease 2019 (Covid-19). Front Immunol (2020) 11:827. 10.3389/fimmu.2020.00827 32425950 PMC7205903

[16] Garvin MR Alvarez C Miller JI Prates ET Walker AM Amos BK. A Mechanistic Model and Therapeutic Interventions for COVID-19 Involving a RAS-Mediated Bradykinin Storm. eLife (2020) 9:e59177. 10.7554/eLife.59177 32633718 PMC7410499

[17] Tisoncik JR Korth MJ Simmons CP Farrar J Martin TR Katze MG. Into the Eye of the Cytokine Storm. Microbiol Mol Biol Rev (2012) 76(1):16–32. 10.1128/MMBR.05015-11 22390970 PMC3294426

[18] Wong CK Lam CWK Wu AKL Ip WK Lee NLS Chan IHS. Plasma Inflammatory Cytokines and Chemokines in Severe Acute Respiratory Syndrome. Clin Exp Immunol (2004) 136(1):95–103. 10.1111/j.1365-2249.2004.02415.x 15030519 PMC1808997

[19] Chan CY Zhao H Pugh RJ Pedley AM French J Jones SA. Purinosome Formation as a Function of the Cell Cycle. Proc Natl Acad Sci (2015) 112(5):1368–73. 10.1073/pnas.1423009112 PMC4321313 25605889

[20] Kondo M Yamaoka T Honda S Miwa Y Katashima R Moritani M. The Rate of Cell Growth Is Regulated by Purine Biosynthesis Via ATP Production and G1 to S Phase Transition1. J Biochem (Tokyo) (2000) J 128(1):57–64. 10.1093/oxfordjournals.jbchem.a022730 10876158

[21] Zhao H Chiaro CR Zhang L Smith PB Chan CY Pedley AM. Quantitative Analysis of Purine Nucleotides Indicates That Purinosomes Increase *De Novo* Purine Biosynthesis. J Biol Chem (2015) 290(11):6705–13. 10.1074/jbc.M114.628701 PMC4358094 25605736

[22] V’kovski P Kratzel A Steiner S Stalder H Thiel V. Coronavirus Biology and Replication: Implications for SARS-Cov-2. Nat Rev Microbiol (2021) 19(3):155–70. 10.1038/s41579-020-00468-6 PMC7592455 33116300

[23] Astuti I Ysrafil. Severe Acute Respiratory Syndrome Coronavirus 2 (SARS-Cov-2): An Overview of Viral Structure and Host Response. Diabetes Metab Syndr Clin Res Rev (2020) 14(4):407–12. 10.1016/j.dsx.2020.04.020 PMC7165108 32335367

[24] DeVito SR Ortiz-Riaño E Martínez-Sobrido L Munger J. Cytomegalovirus-Mediated Activation of Pyrimidine Biosynthesis Drives UDP–Sugar Synthesis to Support Viral Protein Glycosylation. Proc Natl Acad Sci (2014) 111(50):18019–24. 10.1073/pnas.1415864111 PMC4273352 25472841

[25] Mayer KA Stöckl J Zlabinger GJ Gualdoni GA. Hijacking the Supplies: Metabolism as a Novel Facet of Virus-Host Interaction. Front Immunol (2019) 310:1533. 10.3389/fimmu.2019.01533 PMC6617997 31333664

[26] Shin H Kim C Cho S. Gemcitabine and Nucleos(t)ide Synthesis Inhibitors Are Broad-Spectrum Antiviral Drugs That Activate Innate Immunity. Viruses (2018) 10(4):211. 10.3390/v10040211 PMC5923505 29677162

[27] Song J-H Kim S-R Heo E-Y Lee J-Y Kim D Cho S. Antiviral Activity of Gemcitabine Against Human Rhinovirus In Vitro and In Vivo. Antiviral Res (2017) 145:6–13. 10.1016/j.antiviral.2017.07.003 28705625

[28] Hoffmann H-H Kunz A Simon VA Palese P Shaw ML. Broad-Spectrum Antiviral That Interferes With De Novo Pyrimidine Biosynthesis. Proc Natl Acad Sci (2011) 108(14):5777–82. 10.1073/pnas.1101143108 PMC3078400 21436031

[29] Lucas-Hourani M Dauzonne D Jorda P Cousin G Lupan A Helynck O. Inhibition of Pyrimidine Biosynthesis Pathway Suppresses Viral Growth Through Innate Immunity. PloS Pathog (2013) 9(10):e1003678. 10.1371/journal.ppat.1003678 24098125 PMC3789760

[30] Liu Q Gupta A Okesli-Armlovich A Qiao W Fischer CR Smith M. Enhancing the Antiviral Efficacy of RNA-Dependent RNA Polymerase Inhibition by Combination With Modulators of Pyrimidine Metabolism. Cell Chem Biol (2020) 27(6):668–77.e9. 10.1016/j.chembiol.2020.05.002 32442424 PMC7241336

[31] Wang Y Wang W Xu L Zhou X Shokrollahi E Felczak K. Cross Talk Between Nucleotide Synthesis Pathways With Cellular Immunity in Constraining Hepatitis E Virus Replication. Antimicrob Agents Chemother (2016) 60(5):2834–48. 10.1128/AAC.02700-15 PMC4862450 26926637

[32] Quéméneur L Beloeil L Michallet M-C Angelov G Tomkowiak M Revillard J-P. Restriction of De Novo Nucleotide Biosynthesis Interferes With Clonal Expansion and Differentiation Into Effector and Memory Cd8 T Cells. J Immunol (2004) 173(8):4945–52. 10.4049/jimmunol.173.8.4945 15470036

[33] Frohman EM Villemarette-Pittman NR Cruz RA Longmuir R Rowe V Rowe ES. Part II. High-Dose Methotrexate With Leucovorin Rescue for Severe COVID-19: An Immune Stabilization Strategy for SARS-CoV-2 Induced ‘Panic’ Attack. J Neurol Sci (2020) 415:116935. 10.1016/j.jns.2020.116935 32534807 PMC7241359

[34] Frohman EM Villemarette-Pittman NR Melamed E Cruz RA Longmuir R Varkey TC. Part I. Sars-CoV-2 Triggered ‘Panic’ Attack in Severe COVID-19. J Neurol Sci (2020) 415:116936. 10.1016/j.jns.2020.116936 32532449 PMC7241348

[35] Cronstein BN Naime D Ostad E. The Antiinflammatory Mechanism of Methotrexate. Increased Adenosine Release at Inflamed Sites Diminishes Leukocyte Accumulation in an In Vivo Model of Inflammation. J Clin Invest (1993) 92(6):2675–82. 10.1172/JCI116884 PMC288465 8254024

[36] Friedman B Cronstein B. Methotrexate Mechanism in Treatment of Rheumatoid Arthritis. Joint Bone Spine (2019) 86(3):301–7. 10.1016/j.jbspin.2018.07.004 PMC6360124 30081197

[37] Batool S Nawaz MS Mushtaq G Parvaiz F Kamal MA. In Silico Analysis of Glycinamide Ribonucleotide Transformylase Inhibition by PY873, PY899 and DIA. Saudi J Biol Sci (2017) 24(6):1155–61. 10.1016/j.sjbs.2014.11.008 PMC5562383 28855807

[38] Bronder JL Moran RG. Antifolates Targeting Purine Synthesis Allow Entry of Tumor Cells Into S Phase Regardless of p53 Function. Cancer Res (2002) 62(18):5236. 12234990

[39] Corton JM Gillespie JG Hawley SA Hardie DG. 5-Aminoimidazole-4-Carboxamide Ribonucleoside. A Specific Method for Activating Amp-Activated Protein Kinase in Intact Cells? Eur J Biochem (1995) 229(2):558–65. 10.1111/j.1432-1033.1995.tb20498.x 7744080

[40] Meares GP Qin H Liu Y Holdbrooks AT Benveniste EN. Amp-Activated Protein Kinase Restricts Ifn-γ Signaling. J Immunol (2013) 190(1):372–80. 10.4049/jimmunol.1202390 PMC3735359 23180823

[41] O’Neill LAJ Hardie DG. Metabolism of Inflammation Limited by AMPK and Pseudo-Starvation. Nature (2013) 493(7432):346–55. 10.1038/nature11862 23325217

[42] Salminen A Hyttinen JMT Kaarniranta K. AMP-Activated Protein Kinase Inhibits NF-κb Signaling and Inflammation: Impact on Healthspan and Lifespan. J Mol Med (2011) 89(7):667–76. 10.1007/s00109-011-0748-0 PMC3111671 21431325

[43] Xiang H-C Lin L-X Hu X-F Zhu H Li H-P Zhang R-Y. AMPK Activation Attenuates Inflammatory Pain Through Inhibiting NF-κb Activation and IL-1β Expression. J Neuroinflamm (2019) 16(1):34. 10.1186/s12974-019-1411-x PMC6373126 30755236

[44] Spurr IB Birts CN Cuda F Benkovic SJ Blaydes JP Tavassoli A. Targeting Tumour Proliferation With a Small-Molecule Inhibitor of AICAR Transformylase Homodimerization. ChemBioChem (2012) 13(11):1628–34. 10.1002/cbic.201200279 PMC3517147 22764122

[45] Baresova V Krijt M Skopova V Souckova O Kmoch S Zikanova M. Crispr-Cas9 Induced Mutations Along De Novo Purine Synthesis in HeLa Cells Result in Accumulation of Individual Enzyme Substrates and Affect Purinosome Formation. Mol Genet Metab (2016) 119(3):270–7. 10.1016/j.ymgme.2016.08.004 27590927

[46] Mazzarino RC Baresova V Zikánová M Duval N Wilkinson TG Patterson D. The CRISPR-Cas9 Cradsl HeLa Transcriptome: A First Step in Establishing a Model for ADSL Deficiency and SAICAR Accumulation. Mol Genet Metab Rep (2019) 21:100512. 10.1016/j.ymgmr.2019.100512 31516833 PMC6731210

[47] Mazzarino RC Baresova V Zikánová M Duval N Wilkinson TG Patterson D. The CRISPR-Cas9 Cratic HeLa Transcriptome: Characterization of a Novel Cellular Model of ATIC Deficiency and ZMP Accumulation. Mol Genet Metab Rep (2020) 25:100642. 10.1016/j.ymgmr.2020.100642 32939338 PMC7479443

[48] Mazzarino RC Baresova V Zikánová M Duval N Wilkinson TG Patterson D. Transcriptome and Metabolome Analysis of crGART, A Novel Cell Model of De Novo Purine Synthesis Deficiency: Alterations in CD36 Expression and Activity. BioRxiv Preprint (2021). 10.1101/2020.06.23.167924 PMC8291708 34283828

